# Acknowledgment to the Reviewers of *Biosensors* in 2022

**DOI:** 10.3390/bios13010146

**Published:** 2023-01-16

**Authors:** Biosensors Editorial Office

**Affiliations:** MDPI AG, St. Alban-Anlage 66, 4052 Basel, Switzerland

High-quality academic publishing is built on rigorous peer review. *Biosensors* was able to uphold its high standards for published papers due to the outstanding efforts of our reviewers. Thanks to the efforts of our reviewers in 2022, the median time to first decision was 14 days and the median time to publication was 35 days. Regardless of whether the articles they examined were ultimately published, the editors would like to express their appreciation and thank the following reviewers for the time and dedication that they have shown *Biosensors*:
Aashish PriyeLuis CoelhoAbbas MadaniLuis Pastor Sánchez-FernándezAbdel F. IsakovicLuisa PilanAbdelilah BeljebbarLukas FojtAbdollah AllahverdiLukáš MatějovskýAbdul BasitLukasz GorajekAbdul RasheedLung-Ming FuAbebaw Belay JemereMaciej StrzemskiAbedalrhman AlkhateebMadeleine JoubertAbhishek KandwalMadhavi PaliAbhishek KumarMagdalena LaskowskaAbimbola E. OluwalanaMagdalena LigorAbraham FinnyMagdalena MichelAbul B. M. ZakariaMagdalena StobieckaAchuthamangalam B. MadhankumarMagnus FalkAdam GlowaczMahesh P. BhatAdarsh K. GuptaMahmood BaraniAdem OzcelikMahmoud ElbattahAditya VenkatramaniMahmoud MohamedAdrian J. T. TeoMahsa SalehiAdrian Martinez-RivasMahwash MukhtarAdriano MarcheseMajid MalboubiAfrouz Azari AndersonMakusu TsutsuiAgata KowalczykMalgorzata CzichyAgnieszka E. DacaMalkeshkumar PatelAgnieszka KwiatekMallikarjuna GowdaAgnieszka NawrockaManas Ranjan GartiaAgnieszka PodwinManikandan SanthanamAgnieszka Ulatowska-JarżaManohar BhandariAgnieszka WolińskaManoj DiwakarAgnieszka ŻuchowskaMansi GandhiAgnivo GosaiManuel Gutierrez RamirezAgostino IadiciccoManuel Gutiérrez-CapitánAgostino OcchiconeMaomao ChenAhmad Shukri Muhammad NoorMaqusood AhamedAhmed JalalMar AlvarezAhmed M. DebelaMarc VidalAhmed MehaneyMarcello BertoAhmed RadyMarcelo DavidAiguo ShenMarcelo Henrique GehlenAjay Kumar ChinnamMarcelo Saito NogueiraAjay Kumar YagatiMarcin DrozdAjeet KaushikMarcin UrbanowiczAkhil Jabbar MeerjaMarco ErreniAkhilesh Babu GanganboinaMarco LaurentiAkhilesh PathakMarco S. RodriguesAkilarasan MuthumariappanMarco SantonicoAkinrinade George AyankojoMarco ZannottiAlberto Da Nova AraújoMarcos F. S. TeixeiraAlcenísio J. Jesus-SilvaMarcos F.S. TeixeiraAlcilene Rodrigues MonteiroMarcos Vinicius FoguelAlejandro StarkMaria ChernyaevaAleksandar DekanskiMaria De La Cruz Cardenosa RubioAleksandr BugayMaria Del Pilar Taboada SotomayorAleksandra M. BondžićMaria DimakiAleksei Vorob'evMaria EstevezAleksey V. ArseninMaria FreitasAlessandra AmendolaMaría Isabel LucíoAlessandro FantiMaria Leilani TorresAlessandro MengarelliMaría Luisa Fernández-SánchezAlessandro PaghiMaría Moreno-GuzmánAlessandro PerrellaMaria SokolikovaAlex De OliveiraMaria StrianeseAlex Fan XuMaría VitaleAlexander LomzovMarian IonAlexander MuravskyMariangela FedelAlexander SutorMarianna PannicoAlexandra FălămașMarianna V. KharlamovaAlexandra Virginia BounegruMarianne PrévôtAlexei NabokMarie PospíšilováAlexey A PakhomovMariia AndrianovaAlexey N. SkvortsovMarija JozanovićAlexey SeteikinMarina HolyavkaAlexis GalanisMarine WasniewskiAlexis Le FaucheurMario D' AcuntoAlfredo De La Escosura-MuñizMario GutiérrezAlfredo Márquez LuceroMario InclánAli FarmaniMario ProsaAli MarufMario RodríguezAli MirMario SicilianiAli PassianMāris KniteAlinaghi SalariMarisa NicolaiAlisa RudnitskayaMarisela VélezAliya BekmurzayevaMarius VolmerAliza Aini Md RalibMark J. UlineAlmira RamanavicieneMark FrogleyAlok PandyaMarketa VaculovicovaAlvaro MombrúMaros SmondrkAmadeo SenaMarta Gil BarbaAmadeu GriolMarta JarczewskaAmanda M ClarkMarta VeríssimoAmer CharbajiMarta ZanolettiAmer ZakariaMartin HämmerleAmin FatoniMartin KöglerAmir Farokh PayamMartin TaschmannAmir HatamieMartina Medvidović-KosanovićAmirreza AghakhaniMartina PianoAna Díaz-FernándezMartina TorricelliAna L. HernándezMartino GiaquintoAna Maria PutzMaryam DaneshpourAna RoviscoMarystela FerreiraAnahita IzadyarMarzia BianchiAnand Mohan ShrivastavMaša BuljacAnandram VenkatasubramanianMasafumi YanoAnant AgrawalMasahiko SasanoAnastasia I. SolomatinaMasahiro FuruyaAnastasiya SolovievaMasato SuzukiAndjela DraganićMasayori HagimoriAndjelka CelicMasoumeh GhalkhaniAndrás DérMassimiliano GalluzziAndre LopesMatej NjegovecAndrea CossettiniMathias BusekAndrea CruzMathias DolciAndrea Duarte DoetzerMathieu HautefeuilleAndrea MescolaMatin TorabiniaAndreas TsakalofMatteo ParmeggianiAndreea Lorena MateescuMatteo SensiAndrei OkhokhoninMatthew PanAndrei Vasile NastutaMattia BorgarinoAndreja R. LeskovacMauro AngelettiAndres ÖpikMaxim BurkinAndres Quintero-JaimeMazhar SherAndrew J. BonhamMd Abdus SubhanAndrew KirkMd SalauddinAndrew PiperMd. FaiyazuddinAndrey BratovMd. Faruk HossainAndrey NaumovMd. Nizam UddinAndrzej SobkowiakMd. Rabiul AwualAndrzej ZankiewiczMehdi DadmehrAngel Guillermo BracamonteMehmet SenelAngela CapocefaloMei SangÁngeles Peña-GallegoMelike FirlakAngelo Maria TagliettiMelikhan TanyeriAnh Hung NguyenMeltem ElitasAni BaghdasaryanMeng LiuAnimesh SamantaMengjie ChenAnindita SarkarMengmeng LiuAnisa KaurMengyan NieAnkan Dutta ChowdhuryMeysam KeshavarzAnkana KakotiMiaojin ZhouAnna KusmartsevaMichael AppellAnna Maria PappaMichael DM DrydenAnna N. BerlinaMichael G. WellerAnna Renata DembskaMichael KeusgenAnna S. DavydovaMichael O’TooleAnna SankiewiczMichael R. KoblischkaAnna ToldràMichael ThompsonAnpei YeMichailia AngelopoulouAnthimia BatrinouMichal CzerwińskiAnthony Neal WatkinsMichał KotkowiakAnton PopovMichel J. A. M. Van PuttenAnton S. BukatinMiguel A. PelliteroAnton V. NaumovMiguel Ángel LópezAntonella ArenaMiguel Bravo-ZanogueraAntonella CostaMiguel Peixoto De AlmeidaAntonella CurulliMiguel Ponce-VargasAntonella MacagnanoMiguel XavierAntonin PlatilMihaela Badea DoniAntonino FiorilloMihaela BuleandraAntonio Carlos Sant'AnaMihaela GheorghiuAntonio Di BartolomeoMihaela TertisAntonio MinopoliMike PivnenkoAntonio RubinoMikhail BeklemishevAntonio SarolicMikhail Nikolaevich LyulyukinAntony R. ThiruppathiMikhail RyazantsevAnubhav SrivastavaMikhail SinkinAnuj SharmaMikhail Yu. SkripkinAnup PandithMiklós KubinyiAnuradha SinghMilan PaleiAnvay PatilMiloš OgnjanovićAnyanee KamkaewMin LiuAnyang WangMin ParkApurv SaxenaMinghao NieAraceli González-CortésMinghui YangAranzazu VillasanteMingli WangArati SridharanMing-Pei LuArika BridhikittiMingsheng ChenArjun MohanMingxu YouArman Amani BabadiMinli YouArmando Hernández GarcíaMinwei ZhangArnaldo César PereiraMirhojjat SeyediArnaldo Leal-JuniorMiroslav PohankaArnaud BuhotMiroslav ŽivićArthur KopylovMiryam Rincon JoyaArthur McClellandMiyuki TabataArun Kumar TeotiaMo YangArunas RamanaviciusMoe ElbadawiAsanka Sajini YapaMoein Talebian GevariAshish KulkarniMohamad Ali BijarchiAsif NawazMohamed El-AassarAteet DuttMohamed Gamal MohamedAthanasios KoutrasMohamed I. Abdelwahab HassanAtheer AwadMohamed SharafeldinAtilla KormosMohammad AbdolahadAtsushi SakamotoMohammad Al-HatamlehAurelia Cristina NechiforMohammad Al-SayahAurora Hernandez-MachadoMohammad Jobayer HossainAyad AbbasMohammad ZamanAyaz Mohammad AlamMohammadhassan ValadanAymen AlianMohan WangAysu YarmanMohd Khairuddin Bin MD ArshadAzhwar RaghunathMohd Lokman IbrahimBadariah BaisMohit MehtaBalasubramanian MalaikozhundanMohsen AnnabestaniBansi MalhortaMoises Leon-JuarezBanu NergisMonica FiraBao Yue ZhangMonica FlorescuBappaditya ChandraMonica MariniBarbara VercelliMonika KijewskaBasilio VescioMonssef Drissi-HabtiBeatriz GomesMoon Il KimBeatriz Pérez-FernándezMoreno D'AmicoBelal ShohayebMorteza HosseiniBen JohnsonMostafa AzimzadehBénédicte F. JordanMubashir HussainBenedikt LinderMucong LiBenevides Pessela JoãoMuhammad Aniq Shazni Mohammad HaniffBenoît PiroMuhammad AsifBernardo PatellaMuhammad Azmi Abdul-HamidBhabani Sankar SwainMuhammad Hilmy AlfaruqiBhanu ShresthaMuhammad KhanBin GuanMuhammad QaiserBin WangMuhammad RaheelBingdi ChenMuhammad TahirBingxi YanMuhammad ZubairBinita ShresthaMuhammad ZulfajriBiwu LiuMun'delanji C. VestergaardBo FuMun-Ho RyuBo PengMurali Kannan MaruthamuthuBo WuMurat KaynakBo ZhengMurat SerhatliogluBogdan GhermanMurthy ChavaliBogdan TiganoaiaMurugan VeerapandianBollella PaoloMurugappan MurugappanBoon Giin LeeMurugathas ThanihaichelvanBoris KudryashovMustafa Berkan BicerBor-Ran LiMustafa Hikmet Bilgehan UcarBoubié GuelMustafa Serdar OnsesBoya LiuMuthusankar EswaranBranko BabusiakN. NasimuddinBrenda GreenNa NiuBrian BirchNabil SaffajBrian D. PlouffeNada SmigicBrian De La FranierNadia Mahmoudi KhatirBrian Nils LundstromNagaraj P. ShettiBrigitte BruijnsNaglis MalysBriliant Adhi PrabowoNajmeh KarimianBruno Campos JanegitzNan ZhuBruno Vinicius Manzolli RodriguesNandhinee Radha ShanmugamBruno WacogneNaoki NomaBryan James BlackNarendra Pal Singh ChauhanBuddhadev PurohitNaseem AbbasBurcu GumuscuNaseer GolsanamiByoung Choul KimNasr BensalahC. K. SumeshNatalia UgarovaC. ManjunathaNatalia V. KomarovaCaetano Mazzoni RanieriNathan LindquistCamilo A. R. DíazNathan TannerCan HuangNavadeep ShrivastavaCandelaria Martín-GonzálezNavid KashaninejadCansu İlke KuruNazrul Anuar NayanCao-An VuNguyen Quang TrungCarla QueirosNguyen Quoc Khanh LeCarlo C. VersacéNiazul Islam KhanCarlo CamerlingoNiclas SolinCarlos G. JuanNicolò LagoCarlos Andres Galan-VidalNicolò LandiniCarlos Bueno-AlejoNien-Tsu HuangCarlos CaroNikhil BhallaCarlos EscobedoNikola SakačCarlos MarcuelloNikolay GolubevCarlos MarquesNina DimchevaCarlos Torres-TorresNina V. KanevaCaroline Rodrigues BassoNing HuCassamo Ussemane MussagyNirav JoshiCaterina MerlaNirmal MazumderCelia García-HernandezNobuyasu NaruseCésar Fernández-SánchezNobuyuki FutaiChandan SinghNoemí De-los-Santos-ÁlvarezChang LiuNor Azah YusofChang XuNóra AdányiChanghui LiNoureddin SadawiChang-Tong YangNuno Miguel FigueiredoChang-Yue ChiangNur MustafaogluChanKyu KangNurettin SahinerChan-yeong ParkNurgul K BakirhanChao J. LiuNurhidayatullaili Muhd JulkapliChao TanNuria AlegretChao ZhanNurul Yuziana Mohd YusofChathura AbeywickramaNuvia M. SaucedoChathuranga KumarageOana Dragos-PinzaruCheng YangOana HosuCheng ZongOleg I LyaminCheng-Hsin ChuangOleg StukachCheng-Huan ChenOleg VitrikChenghui LiuOleh SmutokChenghui XiaOlexander BarmakCheng-Sheng HuangOlga A. GoryachevaCheng-Te LinOlga KaravaiChengwu ZhangOlga Victorovna NaumovaChenxi YangOlgica NedicChenxu YuOmar Gutierrez NavarroCheol SongOmer SadakChetan ShendeOrnella MaglioChia-Chen ChangOsamu NiwaChia-Liang SunÖzgün Can ÖnderChia-Min YangPablo Luis López EspíChian-Hui LaiPalla GopalChiara SchiattarellaPaloma FernándezChia-Yun ChenPan Kee BaeChien-Wen HsiehPanagiota KoralliChihchen ChenPanagiota PetrouChih-Chin YangPanagiotis KassanosChih-Lang LinPaolo BertoncelloChih-Sheng ChiangPaolo CampioniChii-Wann LinPaolo MaiuriChing-Chou WuPaolo ViscontiChiwan KooParracino AntoniettaChong ChenPasquale DaponteChongwen WangPatrícia M. ReisChris ChapmanPatricia VazquezChristabel TanPatricio LeytonChristian HaritoPatrick D. KumavorChristian L. LaboissePatrick H. WagnerChristiane ThielemannPaul FaragoChristina SiontorouPaul Hsieh-Fu TsaiChristofer BesterPaul VasosChristoph NebelPaul Zavala-RiveraChristophe CaucheteurPaula PintoChristophe VedrinePaula SampaioChristopher PöhlmannPaulina PowroźnikChuandong WuPaulina Sierra RosalesChuanrui ChenPaulo A. Raymundo-PereiraChunsheng WuPavan ChaturvediChuntae KimPavel KhramtsovChunye XuPawan PathakChun-Yeon LinPaweł ChmielarzChunzhi YiPawel KnapkiewiczCícero CenaPawel MarcClémence CheignonPaweł RussekClint HansenPaweł ŚwitCodruta VarodiPedram AzariConcepción GimenoPedro FaiaCong KongPedro Nuno Sousa SampaioConghui LiuPei Ling LeowCongo Tak Shing ChingPeisheng ZhangConsuelo CelestiPeiwu QinCorinne RaveletPeng QiCosimino MalitestaPengcheng XuCristian RavariuPengfei ZhangCristian RotariuPetar KassalCristian ZetPeter BelobrovCristiane KalinkePeter GuttmannCristiano D'andreaPetr DráberCristina AchimPetr SkladalCristina BignardiPetr TůmaCristina CirtoajePhilip GardinerCristina Gutierrez-SanchezPhilippe BrunetCristina TortoliniPhilippe RascleCrystal ChenPhilippe Saint-CricqCsaba HarasztosiPier Paolo PompaCui-Ping FengPierre-Paul VIDALDa-Hua WeiPietro AusielloDaiki TaniyamaPin-Chieh WuDaizong JiPinkie J. EravuchiraDali SunPiotr AugustyniakDamir ZubacPiotr BorowikDan Jeric Arcega RustiaPiotr KamedulskiDan LiPiotr LulińskiDan LuoPiotr MrozekDana Elena PopaPiotr WrobelDanfeng SunPiyanut PinyouDanhui WangPiyush GuptaDaniel PoenarPo-Yu KuoDaniel Quesada-GonzálezPrakash G. KshirsagarDaniela FrankePranab Kumar MondalDaniele VellaPranav GuptaDaniil N. BratashovPratibha GoelDanilo Martins Dos SantosPrativa SahooDaodao HuPreety AhujaDapeng PengPrincipia DardanoDapeng WangPrincy RandhawaDar-Fu TaiPukar MaharjanDarius JegelevičiusPulak BhushanDarius RackusPumidech PuthongkhamDarren TanQi WangDarya KlyamerQihan LiuDavid GiménezQijun HuangDavid MartinezQiliang DengDavid SergeevichevQinqin HanDavid Serrano-GarcíaQinru SunDavide BarrecaQiyuan HeDavide RoccoQun CaoDavide VurroQuoc-Hung PhanDayakar PenumaduRabeay HassanDe GongRachel A. SaylorDean GoldringRadu C. RacovitaDebabrata SenguptaRadu CiorapDebarun SenguptaRadwan NoorDebashis MajiRaewyn TownDebasis MaityRafael Casquel Del CampoDebnath MajiRafael Martos BuoroDeborah PedoneRafael Vargas-BernalDecataldo FrancescoRafal KozdrachDejun HanRaffaele FrazziDenis GentiliRagini SinghDeog-Su ParkRahul GauttamDer-Yuh LinRajalakshmi SakthivelDésirée WünschRajapandiyan PanneerselvamDespina KalogianniRajavel KrishnamoorthyDevarajan BalajiRajeev YadavDevasish ChowdhuryRajesh HaldharDhanjai DhanjaiRajni VermaDhivya KumarRakesh KhilwaniDhruvajyoti RoyRama KomaragiriDi ZhangRama Shanker SahuDianpeng QiRamin Banan SadeghianDiego Leoni FrancoRan ChenDiego MinciacchiRasa PauliukaiteDieter FrenseRasha Mohamed El NasharDiming ZhangRashidah ArsatDimitrios A. KoutsourasRashmi SriramDimitris KouzoudisRavindra Pratap SinghDimitris TsoukalasRaviraj ThakurDina FarrakhovaRaviraj VankayalaDinakaran ThirumalaiRayyan ManwarDing HeRazi-ul HaqueDingqiang LuRebeca Jiménez-PérezDirk RueterRebeca Miranda-CastroDirk TumaReena Vohra SainiDitta UngorRenata BilewiczDmitriy N. ArtemyevRenata KarpiczDmitry O. SinitsynRenata Kelly MendesDmitry TsymbarenkoRenáta OriňákováDmitry V. RoshchupkinRené M. RossiDomenico De BerardisRen-Jei ChungDomenico TricaricoRicardo OliveiraDomin KohRicha PandeyDonato CalabriaRicha SharmaDonato Luna MorenoRichard B. FairDong Kee YiRichard SchasfoortDonglai ZhongRidwan P. PutraDong-Shik KimRiikka PeltomaaDouglas PetkieRishikesh PandeyDrago StrleRitesh Ray ChaudhuriDubravka Švob ŠtracRizwan Aslam ButtDušan MagaRobert AmbroziakDushyant BarpagaRobert DickinsonEdson SantanaRobert SplinterEduardo OsunaRobert ZiółkowskiEduardo ScheerenRoberta D’AurelioEgor VerbitskiyRobertas DamaševičiusEhsanul Hoque ApuRoberto MerlettiEjay NsugbeRoberto RellaEjaz HussainRoberto TermineEkaterina V. KunitsynaRocco CancelliereElena ZavyalovaRocco CitroniEleonora NuceraRodica VoicuEleonora PargolettiRoger C. LoElham DavoodiRohollah NasiriElia GruesoRoland Yingjie TayElias K. SpanakisRoman A. ZakoldaevElisa ContiRoman ElashnikovElisa Mejía-MejíaRoman Grzegorz SzafranElisa MicheliniRomana SchirhaglElisabeth LojouRomana SokolováElisabeth Marion SchneiderRomualdo TroisiElisabetta CanettaRong XiangElisabetta MartiniRongrong LiuElizabeth Natal De-GaspariRoozbeh Abedini NassabElvira GasparRosalinda InguantaEmad F. NewairRosamaria CapuanoEmanuele Luigi SciutoRosario OlivaEmil KhisamutdinovRosella CataldoEmiliano Martínez-PeriñánRoshan DsouzaEmilio Olías-RuízRubira Rafael Jesus GonçalvesEnrico Maria StaderiniRudy LuckEon Soo LeeRui M. BarbosaEric BrouzesRui YanEric De Souza GilRuihua DongEric GutierrezRuilong ShengEric J. WoolfRyoji KuritaEric Yue MaS.S. VermaEsequiel MesquitaSaadat MajeedEsmaeil Doustkhah HeraghSabato MelloneEster GuausSabina LicenEugenio NotomistaSabira SultanaEun Jeong ChoSadaf Bashir KhanEun Seo ChoiSadanand PandeyEunil HahmSaeed OlyaeeEusebiu Ilarian IoneteSagheer OnaiziEva Vargas OrgazSajal ShrivastavaEvando AraújoSalim AlbukhatyEvangelos SkotadisSalman A. KhanEvelio Ramirez MiquetSalvatore DanieleEverson Thiago Santos Gerôncio SilvaSalvatore MicalizioEwa RopelewskaSam KimFabio Di NardoSameer HussainFabio SartoriSami RamadanFabrizio CaroleoSamir Kumar SarkarFahad RashidSamuel GanFahad UsmanSandeep AryaFanli MengSandile Phinda SongcaFarag SallabiSandip GhugeFariba MollarasouliSándor ValkaiFariborz ParandinSangho BokFazio RobertoSanghoon LeeFederica PiccirilliSangmin AnFederico AlettiSang-Myung LeeFederico PratesiSangsoon LimFei LiSaniye SöylemezFei XingSantosh KumarFeiyan CaiSara PimentaFeiyue TengSara TombelliFélix Fanjul-VélezSarada Prasad GochhayatFeng GanSarah De MarchiFeng GuoSaravanan GovindarajuFeng LiSarthak MandalFeng XuSatyendra Kumar MishraFenghua LiSaúl Eduardo Pomares HernándezFengqi GuoSawsan AlmohammedFengxia SuSebastian MaćkowskiFengyu LiSeda KeleştemurFerdinando FebbraioSehun SeoFerenc EnderSeockmo KuFernanda TonelliSeok Jae LeeFernando CardosoSeong Ho KangFernando Henrique CincottoSeonhwa ParkFernando M. Araujo-MoreiraSerban N. StamatinFerri Elida NoraSerena BoninFilip GucmannSerge OstrovidovFlávio Alberto Da Silva FigueiraSergei PiskunovFlavio EspositoSergei TarasovFleming GudaguntiSergey StepanovFlorence LagardeSergio Gómez-GrañaFlorentina-Daniela MunteanuSergio QuinteroFlorina S. IliescuSergiusz ŁuczakFoziyeh SohrabiSethupathi VelmuruganFrancesca LeonardiSeungwon JungFrancesca Luisa ConfortiSevinc KurbanogluFrancesca PetronellaSewook OhFrancesco ArmettaSeyed Saeid MohtasebiFrancesco D'AmicoSeyede Raheleh YousefiFrancesco FerriniSeyyed Alireza HashemiFrancesco PallottiSeyyed Mehdi KhoshfetratFrancisco J. PeñaShabi Abbas ZaidiFrancisco Javier González-CañeteShaghayegh ShajariFrancisco MolinaShahid IqbalFrederic MelinShailabh KumarFrieder W. SchellerShan JiangFucang JiaShangran XieFulden Ulucan KarnakShanmuga SozhamannanFumio OgawaShanshan LiGábor MocsárShanyi ShenGabriel BidauxShaopei LiGabriel Dias RodriguesShaopeng WangGabriel Lucian RaduShaowei WangGabriel NallathambiShao-Yi HouGabriel Ramos-OrtizSharanabasava V. GanachariGabriela Valdés-RamírezSharat Chandra BarmanGaddi BlumrosenSharmili RoyGajendra Pratap SinghShashank K. GahlautGaozhe CaiShashank PandeyGaurab DuttaShau-Chun WangGautam MahajanShazid Md. SharkerGengtao FuShegufa ShetranjiwallaGeorge TsekenisSherif ElkafrawyGeorge-Octavian BuicaShiba DandpatGeorgii KonoplevShichao DingGeorgios N. TsigaridasShih-Chung WeiGeorgios PapagiannisShimshon BelkinGerard C. Van RhoonShin-Ichi OhiraGerardo Gutiérrez-JuárezShiping SongGergana G. NestorovaShiv K. SharmaGesmi MilcovichShivani SathishGhosh DebanjanaShofarul WustoniGibin GeorgeShouhu XuanGihoon ChoiShubhangi ShuklaGiorgia GiovanniniShuhua HanGiorgio BoganiShujie YangGiorgio MalpeliShulin WanGiovanna PalermoShunbo LiGiovanni SignoreShunsuke FurutaniGiulia AbateShuo SuiGiulia CisottoShuo YangGiulia FestaShuyu LinGiulia MassagliaSidharam PujariGiulia RuscianoSijin GuoGiulia SelvoliniSilvia ZorrillaGiuseppe BrunettiSilvie BernatováGiuseppe MaruccioSimona FiorentiniGiuseppe QueroSimone CavaleraGiuseppe Trusso SfrazzettoSimonetta CaponeGiuseppe VaroneSiyi HuGiuseppina Pinuccia CerratoSobhan RoshaniGiwan SeoSobia NiaziGleb TselikovSofia MavrikouGlen KelpSofyan A. TayaGoldie OzaSoheil Abbaspour-RavasjaniGong ZhangSomayeh FardindoostGongke LiSonal ManoharGordon W. IrvineSongbai ZhangGour Mohan DasSongsong LiGovindasamy ManiSonia BahraniGregory BurwellSonia PereiraGreice K. B. Da CostaSooyeon ChoGrzegorz DomekSoumitra SulekarGrzegorz SobotaSourav BhattacharjeeGuangchang PangSpela Zemljic-JokhadarGuangfu WuSpencer SteinbergGuanghui RenSpyridon KintziosGuangtao ZanSrinivas SistlaGuangzhong MaStavros KarkanisGuan-Zheng LiuStavros PoulopoulosGudjon Elvar TheodorssonStefan GassmannGuennadi SaikoStefan Johannes Karl KraussGulam RabbaniStefan KöstlerGuo-En ChangStefan Rahr WagnerGuoqiang ZhanStefan WennmalmGuoyun GaoStefano BellucciGururaj Kudur JayaprakashStefano BonaldoGyudo LeeStefano FornasaroHa Duong NgoStefano LaiHadi HadshemzadehStela GeorgievaHadi MirzajaniStella AslanoglouHafiz Imran Ahmad QaziStephan WalchHafiz-Muhammad-Umer FarooqiStephanie SimonHaibo ZhouStephen James CowlingHaichao WuStephen SemancikHairong WangStergios LogothetidisHairul HamzahSteven MarczakHaisong LinSu Ryon ShinHala MostafaSubhankar SinghaHamid SadabadiSubing QuHamidreza HabibiyanSubir Kumar JuinHanbin MaSubrata MondalHang GaoSuchithra Ashoka SahadevanHangbo YangSudipta BiswasHangrui LiuSujay RayHani Nasser AbdelhamidSujoy GhoshHanne DiliënSüleyman AşırHanquan SuSuman AlishettyHans Peter LangSumant DwivediHan-Sheng ChuangSuna TimurHan-Wei ChangSung-Min KangHao ShenSung-Min ParkHaomin LiuSunipa RoyHarini MohanramSurajit ChatterjeeHarm Van ZalingeSurendra Krushna ShindeHeba AhmedSurovtsev NikolayHeewon JungSusana De Marcos RuizHefeng ZhangSusana SilvaHelena AlvesSushank ChaudharyHelena FelgueirasSutripto MajumderHélène MicheSvetlana NalimovaHerbert Michael HeiseSvetozar MusićHerbert TobiasSvitlana KopylHernando S. SalapareSwati TanwarHiroaki SakamotoSyed Muhammad UsamaHiroyuki FurusawaSyed ShahabuddinHiroyuki TakeiSylvain AchelleHoàng Ly NguyễnSylvain CardinHong ZhouSylvain FeruglioHong-Hao GaoSylwia BalutaHongjie HuSze Shin LowHongju MaoSzu-I YehHongtao LeiSzymon SiecińskiHongwu WangTae-Hyung KimHorn-jiunn SheenTaek LeeHosang AhnTahereh Rohani-BastamiHsiang-Chen WangTailin XuHsin-yi WenTakahiro KuchimaruHsueh-Ju LuTakahiro KusukawaHua ZhuTakahito OhshiroHubin ZhaoTakeshi ShimomuraHugo Cunha Da SilvaTak-Shing ChingHugo Navarro-ContrerasTakumi MitsuhashiHui JiangTakuya TeraiHui LiuTam NguyenHui YanTamal RoyHuidong LiTamara BasovaHulie ZengTamara L. Kinzer-UrsemHung NguyenTamara Lazarevic-PastiHung-Yin LinTamas PardyHyejin MoonTânia MonizHyeran NohTanja ZidaričHyeun Joong YoonTanmay KulkarniHyun Ho LeeTao ChenHyung D. BaeTao JiangHyungseok ChoTao WangIago PereiroTao WuIbrahim AbdulhalimȚarălungă Dragoș DanielIgnacio Ruigómez SempereTaras TurivIgor BuzalewiczTatevik ChalyanIgor MeglinskiTatyana O. PleshakovaIgor OscorbinTayyaba IftikharIgor V KhudyakovTeodor AastrupIhsanullah IhsanullahTeofil JesionowskiIjaz GulTerence T. W. WongIlie BodaleTetsuo SasanoIllyas Bin Md IsaThakshila LiyanageImran ShahThangavelu KokulnathanIn Jung KimThanihaichelvan MurugathasInmaculada Ortiz-GomezTheodoros RampiasInna Skarga-BandurovaThierry LivacheInocencio R. MartinThinzar M. LwinInsoo KimThomas BirngruberIntan Rosalina SuhitoThomas KnopfelIoan BicaTianyu LiIoan TudosaTibor HianikIoana StanculescuTingting YangIoannis VamvakarisTkachenko VolodymyrIrena IvaniševićTolga CukurIrena ZivkovicTomasz KoczorowskiIrwin A. QuintelaTomasz LipińskiIsaac Yves Lopes MacêdoTomasz MajchrzakIsabelle NavizetTomasz RozwadowskiIsao YamaguchiTomasz StrachowskiIslam MosaTomasz SzymborskiIsmail IsmailTomasz WasilewskiIstván CsarnovicsTomoaki TakataIstvan JanossyTomofumi YamamotoItthipon JeerapanTomohiko YamazakiIulia AntoheTomohisa TakayaIvan BratchenkoTomohito SekineIvan R. QuevedoTomonori YasukawaIvan TomasicTorbjörn LedinIvan TorresToshiyo TamuraIvana DrvenicaTrieu NguyenIvana GobinTshimangadzo MunondeIvor FleckTsiakaras PanagiotisIwona DąbkowskaTsung-Rong KuoIwona GrabowskaTsu-Wang ShenIwona Kuźniarska-BiernackaTzu-Pin WangJ. AjayanUkadike UgbolueJ. Sven D. MieogUmamaheswari RajajiJae-Won ChoiUmapathi ReddicherlaJaewook LeeUmberto MichelucciJai PrakashUmit OgrasJaime Javier JuárezUmut VarolJakub ŻurawskiUroš TkalecJalal GhilaneUrszula Lelek BorkowskaJamal M. RzaijUrtė BubnienėJamal UddinUtkarsh JainJames CaliV. V. Raghavendra SaiJan KrajczewskiVaclav PrajzlerJan TkacVaibhav S. KathavateJana FrankováValentina Di MeoJane MurrayValentina HartwigJanggun JoValentina MussiJanire Peña-BahamondeValentina NotarstefanoJanusz SmulkoValentina PreziosiJaroslav FilipValeria DemontisJasmina Casals TerreValerio BiancalanaJasmine Pramila DevadhasanValerio Francesco AnneseJason BeechValerio GiacconeJaspal B. SinghValery V. KalinchukJavier PeñaVan Giang LeJay DesaiVan-Phung MaiJean-Charles BolomeyVasanthan DevarajJean-Pierre HuignardVasilii BorisovJefferson F. D. F. AraujoVasilis NikolaouJeffery DeStefanoVasko JovanovskiJelena MuncanVasyl KravetsJelena SkorucakVasyl MartsenyukJenu Varghese ChackoVeasna SoumJesús Madrigal-MelchorVeera Manohara Reddy YenuguJiacheng HeVenkata Narayana PalakolluJia-Ling RuanVenkatesan Vinoth KumarJian ChenVenkatesh YarraJiandong WuVenugopal Rao SomaJianfei SunVeronica CelorrioJiangbo YuVeronica EspositoJianguo HuVerónica SerafínJianguo WangVessela KrastevaJiankang WuVictor CoelloJianlong JiVíctor De La Asunción NadalJian-Wen WangVictor H. Perez-GonzalezJiao ZhaiVíctor Ruiz-Valdepeñas MontielJiawei WangVictor S. BalderramaJiawei XiangVictoria ShpacovitchJidong WangVictoria V. ShumyantsevaJie LiVilma RatautaiteJie ZhangVincenza CrupiJin ZhouVincenzo MazzaracchioJin ChenVincenzo RandazzoJin LiVinu MohanJina KoVioleta ValchevaJinchao LouVirginia Moreno-GarcíaJing CaiVirginie FelizardoJing PanVladimir BochenkovJing WangVladimir M. MirskyJing WeiVladimir OsipovJing XuVladimir PitschmannJingdong ZhangVladimir ZverevJingfeng HuangVojko MatkoJingqiu LiangVola M. AndrianarijaonaJingxia WangVolodymyr MosorovJingyi LuanVu Khac Hoang BuiJin-Ha ChoiWallace ThoresonJinho YoonWalter CaseriJiong ZhouWalter J. SalcedoJiri BarekWanhe WangJiwoo HongWan-Ting ChiuJoachim WiestWei LinJoan F. AlonsoWei ShaoJoanna BorowiecWei Wu (China)Joanna KosmanWei Wu (USA)Joanna ZajdaWei ZhaoJoão CastelhanoWeichen HuangJoão M. MirandaWei-Hsin TienJoão Paulo WiniarskiWeihua LaiJoaquim C.G. Esteves Da SilvaWei-Jhong JuJoel ImbaudWeijin GuoJohannes BintingerWeijun QinJohn AggasWei-Lung TsengJohn B. WeaverWeimin ChenJohn Bosco Balaguru RayappanWen SunJohn H. MooreWen ZengJohn McGrathWenbo LiuJohn PresleyWendy MeulebroeckJohn S. MitchellWen-Hui WengJohn StanleyWenkai ZhangJohn VakrosWenxiu YangJonathan Sabaté Del RíoWenyan JiaJong Chan HongWenyong LaiJongcheol ParkWilliam PevelerJongho ParkWojciech Rudno-RudzińskiJong-Min LeeWon Chun OhJong-ryul ChoiWonseok LeeJoo Chuan YeoWork Galal Abdelrehim RashwanJoong Ho ShinXanthopoulos KonstantinosJörg OpitzXiandong LengJorge EscorihuelaXiang LiJorge M. S. FariaXiang RenJorge PadrãoXianwen WangJorge Ricardo Mejía-SalazarXiao Dong ZhouJosé A. RibeiroXiao XiaoJosé De Jesús Rangel MagdalenoXiao ZhangJosé J. Castro-TorresXiaodong ShiJosé Joaquín CerónXiaohong ZhouJose L. HuesoXiaojing ZhouJose Luis SantosXiaojun WeiJose Manuel De AlmeidaXiaokun PeiJose Manuel Martin GarciaXiaoliang ChenJosef KollmitzerXiaolin HuangJoseph NdieyiraXiaoqing LvJoseph W. LowdonXiaoshu XuJoshua Van Der ZalmXiaoting JiaJoyce MatsosoXiaoyi LiJuan A. González-VeraXiaozhou LüJuan Arturo SquellaXihui BianJuan Carlos Hernández-GonzálezXin CuiJuan Carlos Martinez EspinosaXinjie ZhangJuan José García-GuzmánXinke LiJuan Luis Pichardo-MolinaXinqin LiaoJuan Manuel GutiérrezXiong DingJuemin YiXiqian JiangJu-Hyuck LeeXiupei YangJui-Cheng ChangXiuyou HanJulianne C. GriepenburgXiyun GuanJulien VieillardXuan Thang VuJulija RazumieneXuanjun ZhangJulija VolmajerXudong JiJulio Bastos ArrietaXue HanJun Hui ParkXue Mei WangJun Ki AhnXuedan WuJun RenXuegang LiJun WangXuejun ZhangJun YangXueqing LiuJun ZhongXuming ZhuangJung Tae LeeXuzhou LiJunkai RenYaiza Gonzalez-GarciaJunren WangYan KazakovJunzhe LouYan LiJuvenal Rodríguez-ReséndizYan V. ZubavichusJyh-Jian ChenYan ZhangKa Lung Andrew ChanYana Roka-MoiiaKagan KermanYang GaoKai ZhaoYang LiKaikai XuYang LuoKaiqiang WangYang RanKaitao LiYanli ZhouKalamullah RamliYann LanoiseléeKaliannan DurairajYao LiangKalloor Joseph FrancisYaofei ChenKalman ImreYap Wing FenKalpana SettuYapa M. Nuwan D. Y. BandaraKamel Abd-ElsalamYaroslav V. BobitskiKamil Reza KhondakarYasunari MatsuzakaKamila MaleckaYasutaka KitahamaKamyar KhoshnevisanYazeed Yasin GhadiKan WuYe ZhangKang CuiYen-Linh Ngo ThiKaori FujinamiYeong-Hwa ChangKari KopraYew Hoong WongKarl WallaceYi HuangKarol StasiewiczYi WuKarthikeyan SekarYi XuKashif ShamimYichao ZhaoKasper EerselsYichi SuKatarzyna RutkowskaYifan BaoKatarzyna WigluszYifan LiuKaterina D. TzimourtaYifan ZhangKaterina TzimourtaYifan ZhuKathryn PflughoeftYi-Fei WangKatsuo KurabayashiYi-Je JuangKatya ArquillaYi-Kuang YenKawsar AhmadYinchen SongKazushige YamanaYing PanKe DuYing TanKeiji MurayamaYingwei LiKejing HuangYiqiang FanKen LeungYiyuan HanKentaro IwamiYohei KurataKerstin LängeYomna AbdelrahmanKetan KotechaYong JiangKevin HoneychurchYong XuKevin M DanielsYong YangKhac-Uan DoYong Zhang (China)Khadine HigginsYong Zhang (USA)Khaled A. SallamYonggang ZhuKhaled Ben KhalifaYongjian NianKhaled Nabil SalamaYongjin SungKhalid ElwakeelYongkun SuiKhurram Shahzad BaigYongliang TangKi Soo ParkYongqin LiKi Tae KimYongsen YuKidong ParkYoon-Hwae HwangKien Voon KongYosi Shacham-DiamandKi-Ho HanYoubin ZhengKiran Kumar TadiYouju HuangKonrad RudnickiYoung Chang JoKonstantin NikolaevYoung-Ho ChoKonstantinos MisiakosYoung-Ki KimKrishna KantYoungwook SeoKrzysztof ŻamojćYouyu ZhangKuangwen HsiehYu HuangKumud Malika TripathiYu ZhangKun LiangYuan CaoKundan SivashanmuganYuan-Fong Chou ChauKun-Lin LeeYuan-Hung WangKyojiro MorikawaYuan-Pang HsiehKyoung G. LeeYuanwei ZhangKyun Kyu KimYubing HuKyung Hyun ChoiYudong ShenLachit DuttaYue GuLaibao ZhangYufeng ZhaoLakshmi Narashimhan RamanaYuhong ZhengLan LiYuh-Shyong YangLars DähneYujing SongLars LindvoldYu-Jui FanLars-Peter KamolzYuki KitazumiLászló BeneYukitomo AraoLászló JanovákYun-Lu SunLaura MicheliYun-Ming WangLaura Rodriguez-LorenzoYunseo KuLawrence H. LeYun-Sik NamLe Minh Tu PhanYunsu MaLeandro SaccoYuqi ChenLehui LuYuqian ZhangLei WangYuri KrakLei WuYurii E. GeintsLei ZhangYury KistenevLenka LhotskaYusin PakLeo AnzagiraZafar IqbalLeonardo PuppulinZaihua DuanLetizia De MariaZaka UllahLevon GevorkovZdeněk FarkaLi Lin (China)Zenkong DaiLi Lin (USA)Zenon LukaszewskiLi XiaZeyi GuanLi ZhengZhan ZhaoLian LiZhangkai ZhouLidia MosińskaZhao HaoLidia Stasiak-RóżańskaZhaohui LiLidong WuZhaowei ZhangLijun WangZhen ZhangLin QiZhenglin YangLina ZouZhenlin XuLingju MengZheyuan ChenLingqian ChangZhigang QiuLingxin ChenZhigang XuLip Ket ChinZhigang YinLiping DuZhiheng XuLiping XieZhihong ZhangLiqiang LuoZhipeng CaiLivia Alexandra Dinu GugoasaZhiwei LuLivia PetrescuZhixing GanLiwei YangZhiyang ZhangLong LiuZhongchao ZhaoLu YinZhuang LiLuca BurrattiZhuangjian LiuLuca De StefanoZhuangqiang GaoLuca TubianaZhuhuang ZhouLucas Hernández-SaraviaZhuo LiuLucian-Gabriel ZamfirZhuofu LiuLuciano BoeselZhuoran MaLucjan JerzykiewiczZidong LiLudek HavranZoltán KókaiLudovica CacopardoZuan-Tao LinLuigi CampanellaZuzana Kozovska


